# Grip Force Reveals the Context Sensitivity of Language-Induced Motor Activity during “Action Words” Processing: Evidence from Sentential Negation

**DOI:** 10.1371/journal.pone.0050287

**Published:** 2012-12-05

**Authors:** Pia Aravena, Yvonne Delevoye-Turrell, Viviane Deprez, Anne Cheylus, Yves Paulignan, Victor Frak, Tatjana Nazir

**Affiliations:** 1 L2C2-Institut des Sciences Cognitives, CNRS/UCBL, Université Claude Bernard Lyon1, Lyon, France; 2 Laboratoire URECA, UDL3, Université Lille Nord de France, Lille, France; 3 Institut de Réadaptation Gingras-Lindsay de Montréal, Centre de de Recherche Interdisciplinaire en Réadaptation du Montréal Métropolitain, Université de Montréal, Québec, Canada; University of Leicester, United Kingdom

## Abstract

**Background:**

Studies demonstrating the involvement of motor brain structures in language processing typically focus on time windows beyond the latencies of lexical-semantic access. Consequently, such studies remain inconclusive regarding whether motor brain structures are recruited directly in language processing or through post-linguistic conceptual imagery. In the present study, we introduce a grip-force sensor that allows online measurements of language-induced motor activity during sentence listening. We use this tool to investigate whether language-induced motor activity remains constant or is modulated in negative, as opposed to affirmative, linguistic contexts.

**Methodology/Principal Findings:**

Participants listened to spoken action target words in either affirmative or negative sentences while holding a sensor in a precision grip. The participants were asked to count the sentences containing the name of a country to ensure attention. The grip force signal was recorded continuously. The action words elicited an automatic and significant enhancement of the grip force starting at approximately 300 ms after target word onset in affirmative sentences; however, no comparable grip force modulation was observed when these action words occurred in negative contexts.

**Conclusions/Significance:**

Our findings demonstrate that this simple experimental paradigm can be used to study the online crosstalk between language and the motor systems in an ecological and economical manner. Our data further confirm that the motor brain structures that can be called upon during action word processing are not mandatorily involved; the crosstalk is asymmetrically governed by the linguistic context and not vice versa.

## Introduction

Traditionally examined by linguists and philosophers, the mental representation of the lexical meaning is now being explored by neuroscientists and cognitive psychologists, generating a large body of sometimes conflicting experimental results and debates (see, for example, [1],[2],[3],[4],[5],[6]). In this context, studies have focused on localizing the neural correlates of word comprehension in the brain ([7],[8],[9]; for a review, see [10]). With solid evidence for the involvement of sensorimotor systems in language processing (for a review, see [11]), the systematic investigation of the interaction between neuronal language systems and sensorimotor structures should provide illuminating clues as to the role of these structures in language processing. Presently, however, the neural crosstalk between language and sensorimotor systems remains poorly understood, in part because most neuroimaging and behavioral studies do not allow the determination of whether motor involvement could be an epiphenomenal, post-comprehension process (e.g., motor intention, motor imagery, and so on) (see [12]) or whether such involvement must be understood as an intrinsic part of the lexical meaning (see [1],[13]). Furthermore, given that on the one hand, fMRI measurements of hemodynamic responses provide poor temporal resolution, and on the other hand, behavioral reaction times (RTs) are measured only after linguistic stimulus presentation, such experimental measures cannot determine whether language-induced sensorimotor activity is a cause or a consequence of lexical-semantic processing. Experimental techniques employed to avoid such temporal resolution problems, such as electroencephalography (EEG) (e.g., [14],[15],[16]) or transcranial magnetic stimulation (TMS) ([17],[18]) can be complex or remote and are not always ecologically sound. Simpler techniques that allow the capture of the online effects of language processing on sensorimotor structures would certainly advance our understanding of the role of these structures in language processing. The goal of the present study is to introduce such a tool while simultaneously assessing the role of the linguistic context on lexically induced motor activity.

At present, only a handful of studies have investigated action-word induced motor-activation in a sentential context rather than in isolation (see [19],[20],[21],[13],[15]). An investigation of the effects of the linguistic context on language-induced motor activation is critical to distinguish among the alternative accounts of observed language-induced sensorimotor activity.

The associative learning model ([22],[17]), which can be considered to be part of the group of embodied theories (see [23]), suggests that links between language and sensorimotor structures develop through simple associative learning. Inspired by the Hebbian theory of learning ([24]), this model proposes that in word learning, the simultaneous activation of language-involved areas and sensorimotor areas involved in action leads to pronounced increases in the synaptic strength between the cells of both areas, generating a functional unity. That is, assuming that “action words” (mostly verbs) are generally acquired and experienced along with the execution of the depicted actions (temporal contiguity) ([25]), this account suggests that the co-activation of the neural networks that include perisylvian language areas and motor areas emerges with experience. Through these shared circuits, the percept of an action word then automatically co-activates motor regions of the brain.

A recent study in which adult participants learned to associate novel words with novel actions confirmed that such co-activation networks can develop rapidly, within a few hours of training ([26]). Thus, this simple associative learning model predicts that brain motor activity induced by an action word should be observed whenever the action word is perceived, independent of the linguistic context in which it occurs (see [17],[27]). Note, however, that words do not consistently trigger the same motor information in all contexts. For example, Hoening and collaborators ([28]) have shown that the neural signature of a concept such as *knife* depends on the feature of the concept that has to be retrieved in the task (e.g., dominant attribute “to cut” vs. non-dominant attribute “elongated”) (see also [29],[30]). If the context can affect language-induced sensorimotor activity, then the simple associative learning account of the word meaning cannot hold.

In contrast with the associative learning model, theories of “Secondary Embodiment” ([12],[31]) proposes that semantic representations are amodal, such that concepts are represented independently of sensorimotor information. These latter models explain language-induced sensorimotor activity though “spreading activation” from regions that code amodal concept representations towards structures that code for sensorimotor representations once the word meaning has already been elaborated (Patterson and colleagues ([31]) suggested the anterior temporal lobe as potential location for such an amodal semantic system). Without denying the possible role of sensorimotor activity in language processing (e.g., enriching word content), a corollary of such models is that sensorimotor systems are not obligatory for the retrieval of the word meaning. Considering that no definitive answer (positive or negative) follows directly from the currently available data (for a review, see [23]), the role of sensorimotor systems in language processing remains unclear. Basic issues, such as a precise description of the crosstalk between language and motor systems, are still missing, and the conditions under which motor structures are recruited during language processing remain to be determined. Answering the question of whether language-induced motor activation is context-dependent or fixed to action concepts will help in evaluating the alternative accounts for the action-language crosstalk outlined above.

In the present study, we explored the impact of sentential negation to assess the degree of context dependency of motor activation in word processing. Sentential negation is a semantic operator that is typically encoded by a specialized morpheme that reverses the truth value of a proposition. Several cognitive aspects of negation have been explored (for a review, see [20]); however, thus far there has been little research on the effects of negation on language-induced sensorimotor activity. Certain studies have suggested that negation could reduce the access to the conceptual representation of the negated items ([32],[33]). For instance, MacDonald and Just ([32]), who compared the speed of word retrieval in affirmative and negative contexts found that negated words (e.g., “no *cookies*”) yielded significantly longer response times. The mechanism underlying this behavioral phenomenon, however, remains unclear. Certain authors (cf. [34], [35]) assume that understanding a negated sentence (e.g., “The door is not open”) requires building an initial representation of the corresponding positive state of affair (e.g., “The door is open”), which is then rejected. According to this view, if the representation of an action word involves neural motor structures, the negated actions should first activate and then inhibit the corresponding motor regions. Currently available neuroimaging ([36],[21]) and TMS data ([37]) on the sentential negation of action terms have shown that negated actions display weaker activation in the cortical motor structures than comparable affirmative ones. Because of technical constraints, however, none of these previous studies allowed the fine-grained temporal analysis that would be required to determine whether reduced motor activity occurs after an initial phase of motor activation or whether negation simply leaves the motor structures less active. Note that although an activation-inhibition picture is compatible with a purely associative learning model, inactivation is not.

The goals of the present study were as follows: (1) to introduce a novel experimental tool, a grip-force sensor (ATI mini-40) that provides the means to make online and direct measurements of the effects of language processing on motor activity ([38]) and (2) to investigate the time course of language-induced motor activation and its sensitivity to the linguistic context by presenting hand-related action words in positive or negative sentences while monitoring how the motor activation component is affected by this syntactic construction. Participants were asked to listen to spoken sentences that contained the action target words embedded within affirmative or negative contexts. Throughout the experiment, the participants held the sensor in a precision grip with their right hand (the thumb, index and middle fingers were in contact with the load cell) such that the grip force signal was registered continuously across a given time interval. A previous study by Frak et al. ([38]) established that this type of sensor can capture subtle grip force variations while subjects listen to single words. In that study, participants listened to words relating (verbs) or not relating (nouns) to a manual action while holding a cylinder with an integrated force sensor. The authors found a change in the grip force when the subjects heard verbs that related to manual action. The grip force increased from approximately 100 ms following the verb presentation and peaked at 380 ms. These observations reveal the relationship that exists between language and grasp and show that it is possible to elucidate new aspects of sensorimotor interaction online.

To attenuate the possibility of mental-imagery effects on motor activation, we avoided the first-person perspective in our sentences and used the third-person perspective instead. It has been shown that first-person process involves mostly a kinesthetic representation of the action, whereas the third-person perspective is much more conducive to visual imagery (see [39],[40]). Moreover, no motor task associated with the linguistic process was required, as the participants were asked to count how many sentences contained the name of a country. This task ensured that potential grip force effects were elicited only by listening to action sentences.

To interpret the time course of language-induced motor activation, we drew on an influential neurophysiological model of spoken sentence comprehension, temporal parameters of which were based on electrophysiological data ([41]). According to this model, information about syntactic structure is formed based on information about word category approximately 100–300 ms after word onset in a first phase. In a second phase (300–500 ms), lexical-semantic and morphosyntactic processes are computed for thematic role assignment. In a third and final phase (500–1000 ms), the information generated in phases 1 and 2 is integrated and reanalyzed. Despite the definition into three discrete time windows, we assume that the processes identified in the model could occur gradually. While observation of language-induced grip force modulation within these different time windows does not automatically imply a causal link between the motor and the language processes, referring to this model will nonetheless allow formulating some clear predictions. Hence, if the motor representation of the action is part of the lexical-semantic representation of the action words, we should expect the following:

- For sentences containing affirmative action words, an enhancement of the grip force as early as 300–500 ms after word onset (c.f. phase 2). This enhancement should continue through the integration phase (c.f. phase 3).
- For negative sentences, either an initial enhancement of the grip force in phase 2, followed by force reduction in phase 3 (this result would confirm the associative learning model), or no modulation of the grip force by the negated action word (this result would refute the associative learning model).

## Methods

### Ethics Statement

All of the participants in this study gave an informed written consent. In accordance with the Helsinki Declaration, the study was approved by the Ethical Committee CPP (Comité de Protection des Personnes) Sud-Est II in Lyon, France.

### Participants

All of the participants were French undergraduate students (18 to 35 years old; mean age = 22.9, SD = 5.4) and right-handed (Edinburgh Inventory definition ([42])), with normal hearing and no reported history of psychiatric or neurological disorders. Twenty-five subjects (including11 females) participated in this study. One participant was eliminated from the analysis due to strong signal fluctuations (exceeding ±0.4 mN) throughout the experiment.

### Stimuli

A total of 115 French sentences served as stimuli (see Stimuli S1). Ten were distractor sentences containing a country name. The data from the trials using the distractor sentences were not included in the analysis. Thirty-five target-action words were embedded into affirmative and negative context sentences, resulting in 70 total sentences corresponding to the two conditions of the experiment: the affirmative condition and the negative condition. All of the target action words were verbs denoting actions performed with the hand or arm (e.g., scratch or throw). Thirty-five sentences containing common nouns denoting concrete entities with no motor associations were used for the purpose of comparison with earlier studies (e.g., [38]). The target nouns and verbs were controlled for frequency, number of letters, number of syllables and bi- and trigram frequency ([43], see Methods S1). Three examples of experimental stimuli are provided in Table 1.

**Table 1:** Examples of the stimuli used in the experiment and their approximate English translations.

Condition	Sentence	English approximate translation
Affirmative action sentence	Dans la salle de sport, Fiona soulève des haltères.	*At the gym, Fiona lifts the dumbbells.*
Negative action sentence	A l'intérieur de l'avion, Laure ne soulève pas son bagage.	*In the plane, Laure doesn't lift her luggage.*
Nouns	Au printemps, Edmonde aime le bosquet de fleur de son jardin.	*In the spring, Edmonde loves the flower bush in her garden*

All critical verbs were in the present tense and in neutral 3^rd^ person. Verbs always occurred in the same sentential position. The sentences were spoken by a male adult. His voice was recorded using Adobe Soundbooth and the recordings were adjusted to generate similar trial lengths using the Audacity 1.2.6 software. Two pseudo-randomized sentences lists were generated from trials; these lists contained uniform distributions of the different sentence types. The two lists were alternated between participants. The mean word duration was 459 ms (SD = 97 ms) for the nouns and 415 ms (SD = 78 ms) for the verbs. There was an interval of 2000 ms between the sentence presentations.

### Equipment and data Acquisition

**Figure 1:**
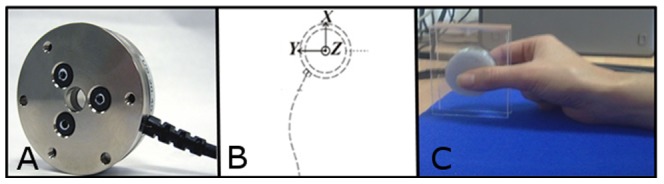
Experimental material. (A) Grip-force sensor (ATI mini-40). (B) A diagram specifying the 3 force axes measured by the load cell. (C) The hand position that was maintained by the participants throughout the experiment.

Two distinct computers were used for data recording and stimulus presentation to ensure synchronization between audio files and grip force measurements (estimated error <5 ms). The first computer read the play-list of the pseudo-randomized stimuli. The second computer received two triggers from the first computer, which indicated the beginning and the end of the play-list. This second computer also recorded the incoming force signals from the load cell at a high sampling rate of 1 KHz. To measure the activity of the hand muscles, a standalone 6-axis load cell of 68 g was used (ATI Industrial Automation, USA, see Figure 1). In the present study, force torques were negligible due to the absence of voluntary movement; thus, only the three main forces were recorded: Fx, Fy and Fz as the longitudinal, radial and compression forces, respectively (Figure 1B).

### Procedure

**Figure 2:**
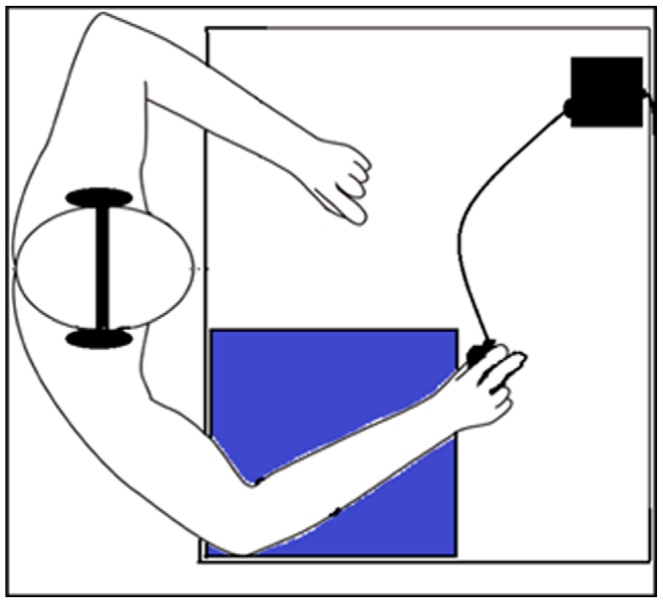
Experimental setting. The participants rested both arms on a padded cushion while holding the grip-force sensor with the right hand in a precision grip (the thumb, index and middle fingers rested on the load cell throughout the experiment).

The participants wore headphones and were comfortably seated behind a desk on which a pad was placed. They were asked to rest their arms on the pad, holding the grip force sensor in a precision grip with their right hand (see Figure 1 and 2). The thumb, index and middle fingers remained on the load cell throughout the experiment. The participants were requested to hold the cell only with the required force to hold it in a nonchalant manner, and not to apply voluntary additional force. The cell was suspended and not in contact with the table. The participants kept their eyes closed for the duration of the experiment. They were instructed to listen to the spoken sentences. Their task was to silently count how many sentences contained the name of a country. To avoid muscular fatigue, a break of 10 seconds was given every 3 min. The total length of the experiment was 12 min.

### Data analysis

Prior to the data analysis, each signal component was pretreated with the Brain Vision Analyzer 2.0 software (Brain Vision Analyzer software, Brain Products GmbH, Munich, Germany). First, a notch filter (>50 Hz) was applied. The data were then filtered at 10 Hz with a fourth-order, zero-phase, low-pass Butterworth filter. Finally, a baseline correction was performed on the mean amplitude of the interval from −400 to 0 ms prior to word onset. The baseline correction was implemented because of a possible global change in grip force during the session (12 min), and because we are only interested in grip-force changes. Thus, we adjusted the post-stimulus values by the values present in the baseline period (−400 ms pre-stimulus to stimulus onset). A simple subtraction of the baseline values from all of the values in the epoch was performed. Thus, the signal effects are based on the assumption that the pre-stimulus is equal to 0 across all participants and conditions. As the participants were asked to hold the grip-force sensor throughout the experiment, a “negative” grip force refers to a lesser grip force and not to the absence of grip force, which is impossible in this context. Only Fz (compression force) was included in the analysis as this parameter was determined to be the most accurate indicator of prehensile grip force. The Fz signals were segmented offline into 1200 ms epochs spanning from 400 ms pre-stimulus onset to 800 ms post-stimulus. The segments with visually detectable artifacts (e.g., gross hand movements) and the trials that showed oscillations of more than ±0.4 mN throughout the segment were isolated and discarded from the analysis. The Fz signals for affirmative action words, negated action words and nouns were averaged for each participant and the grand mean was computed for each condition.

We selected two time windows (i.e. 300–500 ms and 500–1000 ms after word onset) that were identified as critical phases during the processing of words in auditory sentences according to the model of Friederici [41]. Given that the conduction time between the primary motor cortex (M1) and hand muscle is approximately 18–20 ms (estimations using TMS [44]), we added 20 ms to each of these windows, resulting in 320–520 ms for the first time window and 520–800 ms for the second. Because the grip-force values were not normally distributed, non-parametric statistical analyses were performed on the data following three steps: 1) for each condition, the averaged grip force values in the two time windows were compared with their proper baseline (i.e. averaged grip force values over the segment between −400 to 0 ms before target word onset) using Wilcoxon's signed-rank test; 2) for a window that presented significant grip force modulations with respect to the baseline, a comparison between the conditions was performed using Friedman's non-parametric repeated measures comparison; and 3) if the latter comparison was significant, planned comparisons between the two conditions were performed using Wilcoxon's test.

## Results

### Polarity Effects

**Figure 3:**
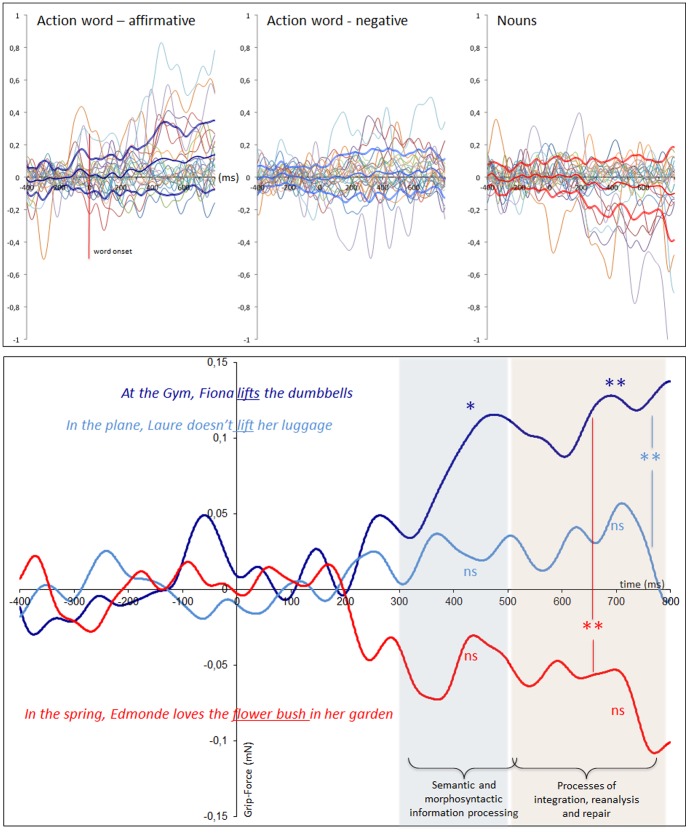
Modulation of the grip-force amplitude as a function of time after target onset. Top panel. Individual data from the 24 participants with mean and standard deviation. Data are plotted separately for the three conditions. Bottom panel. Comparison of data averaged over all participants. Time windows of significant grip-force amplitude regarding the baseline for the affirmative condition are marked by a colored background. For the affirmative sentence condition, testing against the baseline revealed a significant increase in the grip force in both time windows (320–520 ms and 520–800 ms). No significant effects were observed for action words in the negative context or for nouns. A Friedman's repeated measures comparison was significant in the second time window only. Separate Wilcoxon tests for this latter phase showed that the affirmative sentence condition differed significantly from the negative condition and nouns.

Figure 3 plots the variations in grip-force amplitude as a function of time after target word onset for the three experimental conditions (affirmative action, negated action and nouns). The top panel display individual data for the three conditions and the bottom panel compares data of the three conditions averaged over all participants. As is obvious from the figure, until approximately 200 ms after word onset, the grip-force remained comparable and close to baseline for all three conditions. For the action words in affirmative contexts, a steady increase in the grip force (the compression force component of the load cell (Fz)) was subsequently observed, which continued to increase until the end of the recorded segment. By contrast, in the negative sentence contexts, averaged grip-force remained nearly constant at baseline. Finally, the noun targets appeared to cause a drop in the grip-force.

For the affirmative sentence condition, the test against the baseline revealed a significant increase in the grip-force in both time windows [W(23) = 72, Z = 2.229, p = .0258 and W(23) = 42, Z = 3.086, p = .002, respectively]. No significant effects were observed for the action words in the negative context or for the nouns. Friedman's repeated measures comparison was significant in the second time window only (χ^2^ (2,24) = 7.583, p = 0.0226). Separate Wilcoxon tests for this latter phase showed that the affirmative sentence condition (M = 0.11 mN, SD = 0.18) differed significantly from the negative condition (M = 0.02 mN, SD = 0.1) and the nouns (M = −0.06 mN, SD = 0.16) [W(23) = 68, Z = 2.343, p = .0191 for action words in the negative context and W(23) = 58, Z = 2.629, p = .0086, for the nouns].

## Discussion

Through a detailed analysis of grip force modulations, the present study sought to gain a better understanding of the role of motor structures in the lexical-semantic and syntactic-semantic integration processes, by assessing the effects of the syntactic context (affirmation vs. negation) on crosstalk between language and motor structures. With this goal in mind, we used a novel technique that was first introduced by Frak et al. ([38]), which provides the means to capture the temporal dynamics of motor activity during language processing. Our results show the following:

- A significant enhancement of the grip force relative to the baseline when participants listened to words expressing hand actions in the affirmative context. This effect became significant in time windows corresponding to lexical-semantic processes (320–520 ms) and semantic-syntactic integration processes (520–800 ms).
- An absence of grip force modulation for the same action words presented within negative sentential contexts.
- A significant difference in the force amplitude between the action words in affirmative contexts and the two other conditions in the phase of semantic-syntactic integration (520–800 ms [41]).

Hence, although language-induced modulation of the grip force for action words occurs within a time window during which lexical-semantic processes during word processing are occurring ([41],[45]), the offset of this effect by syntactic operations sets limits on the interpretation of the role of this motor activity during language processing.

### Motor structures and contextual word meaning

Our study provides a strong confirmation that the grip-force sensor is a convenient tool that allows the rapid testing of hypotheses about action and language links, which can be confirmed thereafter with more sophisticated methods, as already suggested by the study of Frak et al. ([38]). For anatomical purposes, we insist on the monitoring value of the tool because, although effects on the cortico-spinal motor system can be revealed, this measure lacks the precision necessary to localize the exact locus of the activation-inhibition effects. In the present study, we used this tool to examine the effect of sentential negation on language-induced motor activity at two phases of word processing ([41]). In the first of the two phases (300–500 ms after word onset, or phase 2 in Friederici's model ([41]), during which lexical-semantic and morpho-syntactic information is computed) a significant grip-force amplitude for action words in the affirmative context was found. The enhancement of the grip force in this time window suggests that motor structures could indeed be involved in elaborating lexical-semantic information during action word processing. The absence of such activation, however, for the same action word embedded in a negative context shows that sentential context can prevent the recruitment of these motor structures for the processing of the word. In the second of the two phases (500–800 ms after word onset, or phase 3 in Friederici's model ([41]), which corresponds to the time window within which the different types of previously elaborated information are reanalyzed and integrated), we observed the strongest enhancement of the grip force within affirmative contexts and a significant difference between this and the other two conditions. Before discussing the effect of sentential negation in more detail, we briefly review the results for the nouns. The observed difference in grip-force modulation during the processing of action words in affirmative contexts and during the processing of nouns is consistent with the data reported by Frak et al. ([38]) for isolated words. A straightforward explanation for this difference could be that no motor activity is required for the processing of nouns denoting concrete entities that have no or only weak motor associations ([46]); however, the present results do not eliminate an equally or even more interesting possibility, namely, that the observed difference is a *word category* effect. With well-controlled stimuli, (e.g., as in the study by Olivieri et al. ([46]) that contrasted nouns with or without motor associations to verbs with strong or weak motor associations) the present experimental technique will allow distinguishing between these possibilities.

Our study shows that compared to an affirmative context, negating an action neutralized language-induced motor activity for the target word. This context-dependency started to become evident in a phase during which semantic and syntactic information are computed and becomes strongest in a phase during which different types of information involved in sentence processing are integrated. Our findings clearly oppose a simple associative learning model that assumes that language-induced motor activation results as a consequence of the learning-dependent neural coupling between the perisylvian language areas and the motor areas. As a matter of fact, the associative learning account can be matched to the classic notion of the concept as a referential link between a word and the object or between a word and the action to which it refers ([47]). Yet, words generate meaning individually and as a structured whole in a specific context. Even if the motor structures are activated during the processing of a word form such as “lift”, this activation depends on the relevance of the motor information for the meaning of the word in the given context (cf. *she lifts* vs. *she doesn't lift*). The context dependency of motor activation during action word processing has been referred to as “flexibility” in a review by Willems and Casasanto (see [48] and [30]). Acknowledging the flexibility of language-induced motor recruitment is also acknowledging that concepts might be flexibly tailored to context and that semantic features of concepts are dynamically recruited depending on the given background. Hence, even if shared networks between motor and language structures emerge through associative learning ([26]), perceiving an action word does not trigger activity in these motor regions in a mandatory way.

Although our results oppose the radical model of associative learning, this evidence does not imply a conclusive support for the secondary embodiment account. As we mentioned in the introduction, whether motor structures are necessary for language processing is an issue that cannot be resolved based on the data that is currently available (for a review see [23]). Moreover, the absence of motor activation during the processing of action words in negative sentence contexts is not an argument against a functional role of motor structures in language processing. On the contrary, the context dependency of language-induced motor activity could be indicative of a semantic difference between action words in negative and affirmative sentences. As described in the introduction, the established models of negation (cf. [34]; also [20], [35]) assume that understanding a negated sentence requires an initial representation of the negated state, which is subsequently rejected. The present results do not entirely confirm this assumption because no initial grip force enhancement was observed during the processing of action words in negative sentences. Hence, at least the parts of lexical-semantic representations that might be located in motor structures are not recruited when action words are presented in negative sentential context. Tettamanti et al. ([36]), who investigated the neural correlates of syntactic negation for action-related and abstract sentences using fMRI, showed that in a general, content-independent manner sentential negation was associated with a deactivation of the pallido-cortical areas. On top of these content-independent effects, an increase in activation was observed for affirmative compared with negative action-related sentences in left-hemispheric frontoparieto-temporal regions. These findings are compatible with the present results. In fact, the observed absence of grip-force enhancement for action words in negative sentential contexts in the present study could be the consequence of a general effect of negative polarity on activity in the sensorimotor structures (e.g., repercussions from the deactivation of the pallido-cortical areas), which block the motor lexical-semantic representation of the negated items.

Whatever will turn out to be the neural basis of syntactic negation, in the present results, the flexibility of word meaning implies a rather flexible participation of sensorimotor structures in action word representation. The motor structures are not a fixed part of the network for action word representations; rather, these structures are engaged when context focuses on a particular meaning or purpose. As already noted in a model by Gaskell and Marslen-Wilson ([49]), word form and meaning are not represented as a single processing unit, but are distributed over patterns of activation across phonological and semantic nodes ([49]). According to this model, in speech perception, the retrieval of words within a larger context proceeds by reconciling multiple linguistic levels (including phonological, semantic and syntactic) that must be available to allow rapid and effective word processing. Hence, motor lexical-semantic features should not be systematically available whenever an action word is processed. If the context in which the word is presented supports a motor interpretation, motor activity might become relevant to the meaning representation; however, motor activity can be neutralized by the syntactic operation of negation, as shown by our results and supported by previous studies ([32],[36],[37]).

## Conclusions

### The present findings allow us to draw the following conclusions:

First, the sentential negation of action words does not require a stage of action representation that involves the motor structures. Second, the syntactic operator of negation is crucial for the word form processing at a lexical-semantic level because it modulates the recruitment of certain structures of the motor system during language processing. Third, the motor system is not a mandatory part of the network for “action word” representation, but this system is engaged when motor features of meaning are required, as guided by context.

These findings further demonstrate that the novel experimental paradigm introduced in this study can be used in a notably simple and ecological manner for online studies of the crosstalk between motor and linguistic systems to elucidate new aspects of this interplay.

## Supporting Information

Stimuli S1 **Sentences lists.**
(DOC)

Methods S1 **Parameters of lexical control.**
(DOC)
