# Supplementary material for: Grip Force Reveals the Context Sensitivity of Language-Induced Motor Activity during “Action Words” Processing: Evidence from Sentential Negation
Source: PLoS One. 2012 Dec 5;7(12):e50287. doi: 10.1371/journal.pone.0050287 (PMC3515598; doi:10.1371/journal.pone.0050287)
Supplement: Stimuli S1 — Sentences lists. (DOC) [file pone.0050287.s001.doc]

Affirmative condition

Dans le sentier, Jean scie un tronc d’arbre.

Dans la salle de classe, Bastien jette le papier dans la poubelle.

Pour le diner, Berta râpe du fromage dans ses pâtes.

Pour le barbecue, Abdala sale la viande.

A la piscine, Adela tord la serviette qui est tombé dans l’eau

A la fin du dîner, Abby racle le fond de son assiette.

Dans le magasin, Camille serre le nœud de ses chaussures.

Au bureau, Carlo signe le contrat.

Dans le magasin d’antiquités, Danielle vernit la table.

Dans la rue, David agite la main pour saluer.

A l’institut de beauté, Elena épile les jambes de sa cliente.

Au concert, Elias prend le microphone

Dans la salle de bain, Fabian brosse ses dents

Dans cette caverne, Fanny enfouit les objets précieux

Dans le jardin, Gaël fauche les mauvaises herbes.

A l’entrée de la maison, Gabrielle frappe la porte.

Dans l’atelier, Irène gratte la peinture qui a débordé.

Avec un costume de chat, Ian griffe le sol.

Dans les fêtes d’anniversaire, James jongle avec les oranges.

Dans le métro, Joseph mendie un morceau de pain

Par la fenêtre, Jacqueline montre le chemin.

Le matin, Mathilde peigne ses longs cheveux.

Dans le bar, Anne secoue la bouteille de jus.

Le soir, Vicente arrose les plantes.

En fin de journée, Karine balaye le trottoir.

A l’intérieur de l’avion, Laure soulève son bagage.

Dans la cuisine, Madeleine astique le dos de la casserole

Dans la maison de sa grand-mère, Stéphane colorie les dessins

A la poste, Maël déchire l’enveloppe de la lettre reçue.

A la campagne, Rémi dessine le contour des montagnes.

Dans les embouteillages, Patrick pianote sur le volant.

Dans la douche, Pauline savonne les cheveux de son enfant

Cet hiver, Sabine tricote une écharpe.

A l’école, Salvador découpe des personnages en papier.

Negative condition

Dans la menuiserie, Martin ne scie pas de planche de bois.

Dans le parc, Laurent ne jette pas d’enveloppe par terre.

Dans la cuisine, Lucie ne râpe pas les carottes pour la salade

Pour le piquenique, Timon ne sale pas les œufs durs

Dans la laverie, Celia ne tord pas le linge pour l'essorer

Dans la cour, Alice ne pince pas la main de sa poupée

A la cantine, Elsa ne racle pas l'intérieur de la casserole.

Devant l'église, Lilian ne serre pas la main du futur mari.

Dans la salle de prof, Olivier ne signe pas la feuille d'évaluation

Dans l'atelier d'art, Amandine ne vernit pas le coffre

Pour le petit déjeuner, Yvonne n'agite pas la bouteille de lait

Dans sa chambre, Cannelle n'épile pas ses bras

Au stade, Marion ne prend pas son javelot gris

Devant son miroir, Prune ne brosse pas ses cheveux ondulés

A la plage, Cédric n'enfouit pas ses lunettes dans son sac

A la ferme, Robert ne fauche pas le blé de son champ

A la réunion, Delphine ne frappe pas sur la table avant de parler

Sur un banc, Hector ne gratte pas le dos de son chien

Dans la prison, Yannick ne griffe pas la main du gardian

Au cirque, Philippe ne jongle pas avec des massues

Sur le trottoir, Charles ne mendie pas avec son chapeau

Sur la carte, Eloïse ne montre pas son pays d'origine

En coulisse, Sylvie ne peigne pas l'actrice principale

Dans la batucada, Nicolas ne secoue pas les maracas

Dans le pré, Greg n'arrose pas les tulipes

Dans son manoir, Harry ne balaye pas le plancher

Dans la salle de sport, Fiona ne soulève pas des haltères

Dans sa villa, Lionel n'astique pas la rampe d'escalier

À la crèche, Louise ne colorie pas la tête de son bonhomme

Devant la boite de nuit, Manon ne déchire pas sa carte d'identité

Sur sa toile, Julien ne dessine pas les nuages blancs

Devant son ordinateur, Richard ne pianote pas sur le clavier

Dans son bain, Léo ne savonne pas ses pieds

Sur son fauteuil, Claudia ne tricote pas des chaussettes

Dans les magazines, Luc ne découpe pas des images de maison

Control condition

Dans la montagne, Léonard voit l’aigle qui plane.

Dans le bois, Arthur contemple le hêtre qui date de 1780.

Ce soir, Allan attend son avion pour aller en Écosse

Sur la rive, Frank choisit un canoë pour se promener.

Aujourd’hui, Aurélie découvre la grotte où est le trésor

Dans le ciel, Willy regarde une étoile filante très lumineuse.

Au zoo, Brigitte admire la toison fauve du tigre

De sa fenêtre, Chloé apprécie le mûrier en face de la cabane.

A l’aquarium, Damien observe le requin blanc

A la fin de la promenade, Daniel aperçoit le canyon du regard

A l'unanimité, Raphaël ouvre l'écluse au bateau.

Sur la colline, Aurore cherche le moulin le plus grand.

Par téléphone, Emma réserve la chambre d’hôtel

Chez le notaire, Erick estime le terrain à sa valeur actuelle

Dans le centre commercial, Léa inspecte la vitrine avant d’entrer

Dans la forêt, Emile explore le sentier embroussaillé

Dans le désert, Abdallah vénère son chameau.

Au printemps, Edmonde aime le bosquet en fleurs de son jardin

Dans le parc d’attraction, Thierry visite la caverne du dragon

Pendant la descente, Eléonore pense à la falaise derrière elle.

En Patagonie, Françoise étudie le fameux iceberg géant.

Dans son lit, Véronique rêve d’une licorne qui joue sur la pelouse

A la ferme, Victoria prend soin du pommier de sa grand-mère.

Dans ses rêves, Virginia imagine une prairie paisible.

Deux ans plus tard, Paul se rappelle de la tempête qui a frappé le sud.

Au fond du jardin, Yves a une oseraie très étendue

Au magasin, Sylvain achète un grillage pour son pré.

Quand il fait froid, Baptiste se souvient de la banquise de l’antarctique.

De la réserve, Antonin surveille la barrière de l’entrée.

Dans la maison, Nathan regarde la moquette du séjour.

Dans son appartement, Ophélia partage la penderie avec sa colocataire.

Dans sa maison de vacances, Oscar a besoin d’une rambarde pour les escaliers.

Dans son quartier, Raoul maudit le monument de la place.

Avant de mourir, Ryan lègue le cerisier à sa fille

Finalement, Tara obtient le chevalet le plus haut
