# Supplementary material for: Grip Force Reveals the Context Sensitivity of Language-Induced Motor Activity during “Action Words” Processing: Evidence from Sentential Negation
Source: PLoS One. 2012 Dec 5;7(12):e50287. doi: 10.1371/journal.pone.0050287 (PMC3515598; doi:10.1371/journal.pone.0050287)
Supplement: Methods S1 — Parameters of lexical control. (DOC) [file pone.0050287.s002.doc]

Parameters of lexical control

| **VERBS** | **frequency ranges** | **Letters** | **Syllables** | **Bigrams** | **Trigrams** |  |  |  |
| --- | --- | --- | --- | --- | --- | --- | --- | --- |
| scier | 2,39 | 5 | 1 | 2053,7 | 232,24 |  |  |  |
| jeter | 38,77 | 5 | 2 | 6096,66 | 563,97 |  |  |  |
| râper | 0,23 | 5 | 2 | 1759,06 | 99,85 |  |  |  |
| saler | 0,39 | 5 | 2 | 6306,76 | 471,06 |  |  |  |
| tordre | 2,9 | 6 | 1 | 5814,48 | 338,09 |  |  |  |
| pincer | 2,35 | 6 | 2 | 3354,96 | 277,8 |  |  |  |
| racler | 1,06 | 6 | 2 | 3989,2 | 227,73 |  |  |  |
| serrer | 13,42 | 6 | 2 | 8611,9 | 1106,28 |  |  |  |
| signer | 9,23 | 6 | 2 | 3330,94 | 544,8 |  |  |  |
| vernir | 0,39 | 6 | 2 | 3561,04 | 660,46 |  |  |  |
| agiter | 6,68 | 6 | 3 | 4791,1 | 466,91 |  |  |  |
| épiler | 0,68 | 6 | 3 | 3463,74 | 210,76 |  |  |  |
| prendre | 256,16 | 7 | 1 | 5136,04 | 955,6 |  |  |  |
| brosser | 1,65 | 7 | 2 | 4158,96 | 599,1 |  |  |  |
| enfouir | 1,9 | 7 | 2 | 4528,46 | 371,87 |  |  |  |
| faucher | 2,06 | 7 | 2 | 3594,3 | 728,2 |  |  |  |
| frapper | 21,19 | 7 | 2 | 2929,02 | 354,41 |  |  |  |
| gratter | 4,94 | 7 | 2 | 4152,75 | 744,68 |  |  |  |
| griffer | 1,39 | 7 | 2 | 2372,61 | 141,21 |  |  |  |
| jongler | 0,94 | 7 | 2 | 6503,16 | 289,8 |  |  |  |
| mendier | 1,81 | 7 | 2 | 4827,74 | 908,19 |  |  |  |
| montrer | 66,61 | 7 | 2 | 10581,79 | 2856,44 |  |  |  |
| peigner | 0,81 | 7 | 2 | 3148,86 | 288,22 |  |  |  |
| secouer | 8 | 7 | 2 | 5271,19 | 540,37 |  |  |  |
| arroser | 2,55 | 7 | 3 | 2497,37 | 412,32 |  |  |  |
| balayer | 4,19 | 7 | 3 | 2455,48 | 246,47 |  |  |  |
| soulever | 11,45 | 8 | 2 | 9276,43 | 1187,34 |  |  |  |
| astiquer | 1,16 | 8 | 3 | 3880,11 | 594,07 |  |  |  |
| colorier | 0,32 | 8 | 3 | 5898,38 | 615,55 |  |  |  |
| déchirer | 5,16 | 8 | 3 | 3705,59 | 572,54 |  |  |  |
| dessiner | 9,74 | 8 | 3 | 16644,66 | 3172,44 |  |  |  |
| pianoter | 0,19 | 8 | 3 | 2788,21 | 149,2 |  |  |  |
| savonner | 0,77 | 8 | 3 | 3341,17 | 403,38 |  |  |  |
| tricoter | 1,77 | 8 | 3 | 2900,61 | 193,54 |  |  |  |
| découper | 3,81 | 8 | 3 | 3043,4 | 486,1 |  |  |  |
|  |  |  |  |  |  |  |  |  |
|  |  |  |  |  |  |  |  |  |
|  | 13,9 | 6,8 | 2,3 | 4765 | 629 |  |  |  |

| **NOUNS** | **frequency ranges** | **Letters** | **Syllables** | **Bigrams** | **Trigrams** |  |  |  |
| --- | --- | --- | --- | --- | --- | --- | --- | --- |
| aigle | 9 | 5 | 1 | 3627,42 | 194,03 |  |  |  |
| hêtre | 3,1 | 5 | 1 | 4917,89 | 1667,96 |  |  |  |
| avion | 34,71 | 5 | 2 | 3791,62 | 237,67 |  |  |  |
| canoë | 1,29 | 5 | 3 | 4856,14 | 159,17 |  |  |  |
| grotte | 12,35 | 6 | 1 | 4013,74 | 424 |  |  |  |
| étoile | 32,42 | 6 | 2 | 3838,43 | 227,65 |  |  |  |
| toison | 3,42 | 6 | 2 | 8015,57 | 1263,44 |  |  |  |
| mûrier | 0,35 | 6 | 2 | 2879,87 | 288,44 |  |  |  |
| requin | 1,29 | 6 | 2 | 3741,16 | 159,44 |  |  |  |
| canyon | 0,58 | 6 | 2 | 4775,36 | 98,74 |  |  |  |
| écluse | 1,9 | 6 | 2 | 1672,2 | 184,56 |  |  |  |
| moulin | 14,52 | 6 | 2 | 11156,36 | 676,74 |  |  |  |
| chambre | 231,23 | 7 | 1 | 3132,07 | 1005,93 |  |  |  |
| terrain | 61,87 | 7 | 2 | 4704,97 | 969,53 |  |  |  |
| vitrine | 11,42 | 7 | 2 | 4474,5 | 532,88 |  |  |  |
| sentier | 16,39 | 7 | 2 | 7737,99 | 1324,38 |  |  |  |
| chameau | 3,52 | 7 | 2 | 3897,52 | 1058,85 |  |  |  |
| bosquet | 1,77 | 7 | 2 | 2248,54 | 599,46 |  |  |  |
| caverne | 4,9 | 7 | 2 | 2999,25 | 412,82 |  |  |  |
| falaise | 9,74 | 7 | 2 | 4701,2 | 798,53 |  |  |  |
| iceberg | 0,77 | 7 | 2 | 1188,83 | 31,97 |  |  |  |
| licorne | 1,1 | 7 | 2 | 2571,27 | 397,42 |  |  |  |
| pommier | 5,35 | 7 | 2 | 7236,32 | 1767,02 |  |  |  |
| prairie | 9,29 | 7 | 2 | 6623,51 | 663,49 |  |  |  |
| tempête | 17,42 | 7 | 2 | 2971,79 | 562,34 |  |  |  |
| oseraie | 0,29 | 7 | 3 | 2658,04 | 311,02 |  |  |  |
| grillage | 5 | 8 | 2 | 1899,71 | 319,69 |  |  |  |
| banquise | 1 | 8 | 2 | 3695,2 | 282,94 |  |  |  |
| barrière | 12,48 | 8 | 2 | 4371,53 | 391,89 |  |  |  |
| moquette | 7,97 | 8 | 2 | 2650,77 | 339,62 |  |  |  |
| penderie | 1,39 | 8 | 2 | 4693,45 | 765,49 |  |  |  |
| rambarde | 1,32 | 8 | 2 | 1494,19 | 156,91 |  |  |  |
| monument | 8,61 | 8 | 3 | 6753,73 | 1246,29 |  |  |  |
| cerisier | 1,68 | 8 | 3 | 6076,53 | 479,05 |  |  |  |
| chevalet | 3,35 | 8 | 3 | 2509,06 | 544,19 |  |  |  |
|  |  |  |  |  |  |  |  |  |
|  |  |  |  |  |  |  |  |  |
|  | 15,2 | 6,8 | 2,0 | 4245 | 587 |  |  |  |

| FQ OCCU | F(1, 142)=.0006; p = .9798 |  | 0,90 |
| --- | --- | --- | --- |
| SYLL | F(1, 142)=1.7373; p = .1897 |  | 0,09 |
| BIGR | F(1, 142)=1.8422; p = .1769 |  | 0,39 |
| TRIG | F(1, 142)=.5321; p = .4670 |  | 0,76 |

|  | NOUNS | VERBS |
| --- | --- | --- |
| FRQ | 13,92 | 15,22 |
| LETT | 6,80 | 6,80 |
| SYLL | 2,26 | 2,03 |
| BIGR | 4765 | 4245 |
| TRIG | 629 | 587 |
